# The French Practice of Medicine: Being a Translation of L. J. Begin’s Treaties on Therapeutics, &c., &c.

**Published:** 1831

**Authors:** 


Art. X.—*The French practice of medicine: being a translation of L. J. Begin's Treatise on Therapeutics, &c. &c.* By Xavier Tessier. New York: E. Bliss, 1829.

The work before us is not, in itself, sufficiently meritorious to excite any particular attention. It professess, however, to be a regular and systematic exposition of the therapeutic principles of the "physiological doctrine;" and, as such, it affords us occasion for some remarks on the value and tendency of the leading tenets of this school. M. Begin is a thorough-bred Broussaian. He enters upon his task with a most determined contempt for every thing that has been said, or may be said, in opposition to the arguments and dogmas of his pathological creed. He is not behindany one of his *confreres*, in the vehement zeal, dictatorial assertion, and self-applauding strains of triumph and superior sagacity, so common with the advocates of this doctrine. The epithets of "empiric," "charlatan," "ignoramus," and many more equally liberal terms, are freely, and almost as a matter of course, applied to those contumacious beings, who still persist in rejecting the Broussaian pathology. There is no alternative. Either you must embrace the doctrine, or content yourself with being considered as an egregious blockhead. Like the ambitious impostor of Mecca, the disciples of the physiological school, seem to think that the best way to propagate their tenets, is to knock out the brains of the faithless, or what is the same thing, to assert that they have no brains at all. According to these ardent gastro-enteritic pathologists, medicine was a mere chaos—*rudis indigestaque moles*—over which the genius of ontological darkness brooded until the sun of *val de grace* rose and vivified the dormant germ of medical philosophy. All that we, and others equally simple, fondly regarded as the accumulated treasures of medical science—all the facts, and principles, and practical maxims, which the experience and common sense of the profession, from the days of Hippocrates down to our own time, have stamped with the seal of usefulness and truth, we now learn from the mouth of the oracle himself, are, verily, nothing but "*vieilles erreurs*"—"*anciens prejuges*"—"*bigarrures*"—"*un amas informe de verites et d'erreures*"—a most lamentable accumulation of absurdities, errors, and prejudices. We might, indeed, be sorely mortified in thus hearing that all our imagined acquirements in medical science, gained by a long course of toilsome study and enquiry, turns out, after all, a mere mass of nonsense and error—did we not hear from the same *tripod*, the happy annunciation of the speedy advent of a medical millenium; to be consummated in the demolition of every thing that had been done in medicine "*jusqu a l'epoque de la doctrine physiologique*, and the universal adoption of "*cette doctrine bienfaisante*," which, we are told, is now established on "*des bases inebranlables!* Not that the direful box of Pandora is to be closed; but its former brood of distempers—the *nova cohors febrium* are, hereafter, by the absolute decrees of the "new doctrine," to rush out on their errands of destruction, under no other form than of *gastro-enterite;* which is as nothing, almost, against the potency of leeches, gum water ,and emollient enemata.

If simplicity is to be regarded as a paramount recommendation in medical systems, the Broussaian doctrine is most assuredly incomparably excellent. Let us suppose a student to pass round the ward of an hospital under the guidance of a disciple of this school. Pray, what disease is it under which this poor fellow is suffering? *Ma foi!* it is what the "*Purgons*" and empirical ontologists call a dropsy, but *c'est une veritable gastro-enterite.* What is the matter with this patient? This man's disease, Sir, is preposterously called rheumatism. These inepts do not see that it is a flagrant *gastro enteritis.* This, *Monsieur le Docteur*, I take to be a case of intermitting fever, *n'est ce pas?* Intermitting fever *au diable!* No; intermitting fever is a pathological abstraction. I tell you 'tis *gastro enterite!* Let us move on; here is a case of small-pox? *Encore une sotte abstraction ontologique!* no, no, 'tis *gastro-enterite!* Is this case not a well-marked typhus fever? Ah, typhus! an ontological monster!

*tenez, ne voyez vous pas*—how he grins and starts when I thrust my fist into his epigastrium—'tis *gastro enteritis, tres prononce*. But tell me—are all the rest in this ward laboring under gastritis? that epileptic—and that child with measles—and this unfortunate maniac? *Oui, oui, tout le monde!* Blessed simplicity! happy generation of students! ye that now may acquire a knowledge of the nature of nearly the whole catalogue of human maladies, in the time that was, formerly, scarcely sufficient for becoming acquainted with the essential phenomena of a single disease!

But it is time to turn to our author. After making a few observations on the "relations existing between therapeutics and the other branches of medicine," of no particular interest, the author devotes the second chapter to the consideration of the "*medicating powers of nature*." All must admit, he observes, that the animal organization is endowed with a disposition tending to expel morbific causes, to resist their impressions, and to restore within itself the harmony of its functions." This sanative tendency is not, however, to be considered as the operation of an independent principle, a TO THEION or *archaeus*, which, with a kind of intelligent guardianship over the welfare of the animal economy, sets on foot certain actions, designed and especially adapted to oppose, and finally, in many instances, to subdue diseases. Whatever spontaneous actions or results may occur in diseases, calculated to give them a favorable tendency; or whatever resistance the living organization may make to the influence of morbific causes, or to the extension of maladies, must be ascribed to the mutual support, which, by a pervading sympathetic association the various organs of the living system necessarily give to each other, and to the inherent tendency in vital matter to resist, to a degree, and often to triumph gradually over the impressions of injurious agents. Our author observes, however, and very correctly, that the practice formerly so common, and favored, even at the present day, by some, of patiently abstaining from the employment of active remedial means, lest these sanative tendencies should be interrupted, and of confining the curative efforts, to the application of remedies calculated only to support and aid nature in her endeavors to cure the disease, must necessarily, in many cases, lead to the most ruinous consequences. The question, how far we ought to submit diseases to the operations of this restorative tendency of the organization, and especially to what extent we should make the apparent salutary actions of the system the guide of our remedial measures, has, indeed, given rise to abundant discussion. Upon this point, we may observe, that he who always at once makes a bold and uncompromising attack on the disease, without allowing some part of the work to be performed by the physiological efforts of the system; who walks forth armed, at all points, with the most potent weapons of his craft, determined to hold no parley with disease, will be as likely to do mischief, as the practitioner who watches with philosophic calmness over his patient for the critical concoction of morbid humors, or thinks himself unwarranted to prescribe any but the mildest remedies, conformably to the sanative efforts of nature. The former class of practitioners resemble the mariner who would expect to conduct his vessel safely over the traceless ocean, without paying any regard to the indications held out by nature of the approach of tempests and calms; whilst the latter may be compared to the sailor who would expect the winds and waves to waft him to his destined port, without plying his rudder or shifting his sails.

Much advantage may unquestionably be derived from a careful observance of the apparently salutary movements of the system; and yet, a vigorous and well directed blow at the disease in its commencement, will, in most instances of acute affections, determine the contest in favor of the patient. "When in acute diseases," says our author, "indications of a salutary effect are exhibited, or when the irritation is carried to the kidneys, the skin, or the lower extremity of the digestive canal, it is evident that a judicious physician will confine himself to assisting nature in that process. If no intensity exists, and if the patient is so situated that the disease can disappear spontaneously we are undoubtedly to abstain from the use of all energetic measures. Yet in such a case the physician is not inactive; he observes, he operates, he modifies the organism, by directing and promoting the tendency to recovery."

The third chapter is devoted to observations: "*On the basis of curative indications.*" In the commencement of this chapter, the author makes the following well expressed and very correct observation. "With the aid of an extensive erudition and a subtle mind, it is possible to advocate and support all sorts of opinions, if not by means of sound reasoning, at least in a manner calculated to dazzle and subdue weak and superficial observers. The case is altogether different at the bed-side of the patient. In the presence of nature it is much less necessary to discuss than to act; and clinical results will plead more eloquently in favor of the skilful practitioner than the most elaborate discourses." The author fixes himself very comfortably on the vantage-ground of this truism, and regards those who oppose the physiological doctrine, as, at best, but ingenious sophisters, and cunning disputants, who are liable to be overwhelmed with embarrassment and discomfiture in the actual presence of nature, when at the bed-side of the patient. "Some modern empirics" he says, "have dared to assert that we ought to direct our attention solely to the most prominent and dangerous phenomena of morbid affections. But who does not see that *the medicine of symptoms* is absurd in principle and pernicious in its consequences." We are not among those who hold that our remediate efforts should always be especially directed against the more prominent symptoms of maladies, without a particular reference to the immediate causes upon which they depend; and we believe that there are few if any respectable physicians, who would either advocate or adopt in their practice, an entire reliance upon combating *symptoms*, to the total disregard of the local or general morbid conditions of which they are the external manifestations. Not unfrequently, however, we have no other guide or justifiable grounds for prescribing remedies, than the symptoms of the disease before us; and experience warrants the assertion, that in most diseases of an accute character, prescribing for the symptoms is attended with all the success that can be expected from remedial management. Most assuredly, where we can ascertain, that this or that organ or structure is the seat of a latent inflammation, an advantage will be derived from directing our remedies more immediately to the disordered part. But in what, after all, does the boasted treatment for the *causes* of *acute* diseases differ from the "medicine of symptoms" as it is, *gallice*, called. A patient suffers with violent, lancinating pain in the side; attended with a full and vigorous pulse, hot and dry skin, cough and bloody expectoration, costiveness, &c. The "modern empiric who dares to assert that we ought to direct our attention solely to the symptoms," bleeds his patient, in the first place, to diminish the action of the head and arteries; he gives him demulcents to abate the cough; prescribes a laxative to remove the costiveness, and he cups, leeches, and *blisters*, to relieve the pain in the chest. Although he thus meets the symptoms, he is not ignorant that there is *inflammation* in the chest; and he knows from experience, that in opposing the symptoms effectively, he necessarily reduces the inflammation. And what does the "*Medicin philosophique*" do? Why, he bleeds, and leeches, and blisters, and gives demulcents, not to remove the symptoms, but to cure the inflammation! But the true point of tenderness with the Broussacan physicians, is the application of the "*medicine of symptoms*" to general fevers. To treat fevers of this kind according to the symptoms, is, in their estimation, rank empiricism. Such fevers must, according to their creed, be treated as cases of mere *gastro-enteritis*, without any regard, or, at least, but a very subordinate one, to the general symptoms. Whether any *manifestations* of gastro-enteric inflammation exist or not, makes no difference, for they have agreed that it ought to exist, and it is therefore ordained that leeching, cupping, and fomentations over the abdomen, and mucilaginous drinks internally, is the only legitimate and philosophic mode of combating these fevers. As gastro-enteritis, is, according to this school, the essential malady, of, perhaps, ninety-nine in a hundred, of our ordinary diseases, the practitioner need not trouble himself in the investigation of symptoms; not even a thrust of the fist or fingers into the epigastrium, (unless it be to see how much pain can be given,) for he knows, beforehand, that the seat of the disease is in the mucous membrane of the intestinal canal, and that is all he need care about, as being the most certain indication of the most rational course of treatment possible. "There is no practitioner," says our author, "who, having practised according to nosology, has not frequently treated fevers, pains, hypochondriasis, &c., without suspecting the existence of gastro-intestinal irritation." Truly, though no nosologist, we are among this very class of practitioners; and we do confess that we have treated many instances of the forms of disease, here mentioned, without suspecting the existence of gastro-enteric irritation, as the proximate cause of the morbid phenomena. We are consoled, however, by the reflection, that we have had a great many very clever companions on this high-way of error and empiricism; and we must own, that, like most other successful offenders, we feel exceedingly loath to mend our ways in this respect. This whole chapter is indeed little else than a vehement rhapsody against the practice of consulting the symptoms of diseases as the basis of our therapeutic indications. To a mind not warped by the ardor which seems to enkindle the disciples of the physiological school, the extravagant denunciations against those who adopt a different pathological creed, is, of itself, no inconsiderable indication of the weakness of their system, and their own consciousness of the difficulty of defending it against the solid weapons of reason and observation.

We agree, nevertheless, with our author, that "the nature of the disease, or that sort of vital modification, upon which the external morbid appearances depend, is to be the constant object of all therapeutical indications." This will be admitted by the most strenuous advocate for managing diseases according to the indications furnished by the symptons; for what can we ascertain concerning the "nature of diseases, or the vital modifications," which constitute their essence, but what is obtained from a contemplation and due estimate of their symptoms! When a disease is before us, we ca.efully examine all its obvious phenomena, and from these, determine, whether it is febrile, inflammatory, nervous, spasmodic, &c.; and from the same source we decide, in what organ or structure the morbid actions principally exist. These then, namely, the nature and seat of the disease, if it be possible to come to any definite conclusions on these points, ought to be the constant objects of our therapeutic efforts; for it is only by removing these morbid conditions that we can hope to cure the malady. The general bearing of the curative indications, certainly rest on the ideas we form of the nature of the malady; yet the particular symptoms of the case are to be consulted, as guides for modifying and managing these indications to the greatest advantage of the patient. If we were informed that a patient labored under inflammation of the lungs, we would at once say that the indication is for an antiphlogistic treatment. But who would venture to prescribe the extent of the blood-letting, purging, &c., necessary, without having an opportunity of examining and watching the symptoms and cause of the disease, as important, nay, indispensible indicia for the fulfilment of the main indication drawn from the general character or nature of the malady.

In the fourth chapter the author discusses "*the circumstances which contribute to modify indications in the treatment of diseases.*" These circumstances relate to the age, temperament, sex, profession, habits, constitutional vigor or debility of the patient, and to the causes, seats, intensity, and periods of diseases. Each of these topics is treated of under a separate head at considerable length. In relation to the peculiar constitutional habit of very young subjects, the author makes the following observation, which is founded on correct observation and worthy of especial attention.

"It is important to remember, in the observation or treatment of diseases in young subjects, that their sympathetic functions are more active and susceptible, than in adults. Owing to the predominance of their nervous system, the irritations with which they are affected, may easily be complicated with unusual phenomena, and with violent over-excitements of the brain and its membranes. The stomach and intestines being then endowed with a greater vital energy, and exposed to the most powerful causes of disease, their irritation is very frequent, and generally forms a complication of almost all phlegmasiæ. At the first periods of life, irritations are more rapid in their progress; the secretions furnished by irritated mucous membranes, have a singular tendency to coagulate into anomalous membranes, in a manner to obstruct the pasages in which they are situated. In children derivatives act with more energy and efficacy than in adults, owing to the greater susceptibility of the nervous system, and to the facility with which their irrritations can be displaced.

In relation to the *temperaments*, M. Begin observes, that they consist in peculiar modifications of the animal economy, depending on the relative activity and development of the nervous, lymphatic or sanguiferous systems: or upon the predominance of one organ or set of organs. The former modifications are universally diffused throughout the organization, extending to every part where the system of structures, upon whose predominance they depend, are found; whilst the latter variety of physiological modifications act only through the medium of sympathy on other parts of the system.

"The predominance of actions which is limited to one or more organs, such as the heart, the lungs, the liver, the stomach, the genitals, the muscles, the articulations, &c., has the constant effect of converting those organs into as many centres, towards which all the sympathetic irritations converge, and in which they make their appearance. Stimulating agents, although they may operate on the remote parts, never fail to be felt by the most sensible and irritable organ. Thus in subjects where the stomach and kidneys are the seat of the predominant vital action, a violent moral excitement, a damp cold, or any other cause of a similar nature, will be sufficient to create a more or less acute gastritis or nephritis."

The author observes, more justly, that mere sympathetic irritations of an organ or structure, are by no means to be regarded of little consequence, and of easy cure. These local sympathetic irritations, though at first, mere functional derangements, with an altered state of the sensibility and irritability of the part, may soon degenerate into unequivocal inflammation.

and its various consequences. Post mortem examinations have shewn, that the sympathetic irritation of the brain, from a primary irritation or inflammation of the gastro-intestinal mucous membrane, may, even in a few days, pass into a state of fatal arachnitis, and "give opacity and thicknes to this membrane." "The same takes place in sympathetic irritations directed on the pleura, the peritoncum, the synovial membranes, the lungs, and all the other parts." "I believe," says the author, "we may establish, as a fixed principle, that whenever those irritations have acquired a certain degree of intensity, and have existed for some days, they require the same treatment by emollients, &c., as if they had existed independent of any primary affection seated elsewhere. Thus, we are enabled to pursue the irritation wherever it makes its appearance: we wear it out, as it were, and extinguish it in whatever place it may resort to, and from whence it may irradiate to other parts."

In relation to the constitutional or accidental *debility* of certain organs in the system, the author makes the following judicious remarks. "In the generality of cases, at the same time that those organs are weakened, others enjoy an increase of excitement and energy, in such a manner that the economy becomes the seat of an unusual repartition (inequilibrium) of strength, which must be diminished by a decrease of the predominance of action in the over-excited parts, and increased in the others. In such cases, the physician must not consider exclusively the one or the other of these conditions; he must embrace the whole of the economy and restore the harmony between its different parts, giving strength to the latter and abstracting it from the former." An augmented or preternatural degree of excitability is always a consequence or concomitant of general debility. The equilibrium of excitement is easily destroyed, and exciting causes are much less easily borne, at the same time that violent local irritations are especially apt to be rapidly developed.

In relation to what Dr. Rush denominates "suffocated excitement, or what is more generally called an oppressed state of the vital energies, we cannot agree with the author in ascribing it to an "excess of constitutional vigor." This oppressed condition of the vital powers is characterised by a sense of uneasiness and oppression in the thorax and abdomen, an indescribable feeling of anxiety, great prostration of strength, and a small, weak, but usually firm pulse. According to our own views, this condition is the result of general plethora, in conjunction with weak powers of vital resistance giving rise to the accumulation or congestion of blood in the large internal vessels of the thorax and abdomen. Oppressed action of the heart and arteries, is, however, not necessarily, or indeed, often preceded by any manifestations of actual *debility*. An individual may be constitutionally endowed with weak powers of vital resistance to the influence of exciting causes, and yet manifest no actual debility or feebleness of the vital functions. Oppressed or suffocated excitement of the heart and arteries, almost invariably occurs in the commencement of fevers, before the energies of the system have been reduced by the influence of the disease. So far as our observations enables us to judge, we should say, that the state of oppressed action is never observed except immediately after the primary stage of oppression—the chilly stage—which almost universally ushers in the febrile excitement of acute diseases. When the system is plethoric, and not constitutionally endowed with strong powers of vital resistance, the oppression of the vital energies during the initial or chilly stage of the fever, will be apt to be so great as to prevent the heart from propelling the blood, which is strongly congested in its chambers and in the large internal vessels, to the periphery of the circulation; its efforts will be oppressed, and a state of universal debility and inactivity will be the consequence. The only mode of relieving this state of oppression is to lessen the mass of the blood, and to solicit the circulation from the internal vessels. The late Dr. Rush—*clarum et venerabile nomen*—in his usual happy manner of illustration, compared this state of vital oppression, to a man, in health, pressed to the earth with a burden too heavy to bear. To enable him to rise and walk off wth his load you must remove a portion of it from his shoulders: and in the same manner to enable the heart to resume its regular degree of action when thus oppressed, you must take off a portion of its load by venesection and suitable revulsives. It is of the utmost degree of importance, in a practical point of view, to distinguish this state of vital oppression occurring in the early stages of febrile diseases, before the energies of the system have suffered any actual impairment; and that state of oppression and congestive prostration which we sometimes meet with in the advanced stages of fevers of a low or typhoid character. The heart is liable to a sudden diminution of its powers by sympathizing with a nervous irritation primarily located in the alimentary canal. When this occurs the pulse becomes extremely feeble, the muscular system suffers great prostration; the countenance becomes pale and expressive of intense anxiety; the skin is cool and clammy; the sight obscure and confused; the patient manifests an extreme degree of restlessness by constant sighing and jactitation. This state of oppressed excitement is apt to occur in some forms of puerperal fever, and occasionally in the advanced stage of bilious and typhoid forms of fever. Blood-letting here would almost inevitably prove fatal, yet an active *cathartic*, followed by opiates, will often bring on an immediate development of the vital actions.

Actual debility is very rarely universal in febrile and other diseases. There are always some organs or structures in which there is an over-excitement, at the same time that others will be in a state of exhaustion or great feebleness of action. Hence in the treatment of fevers, our principal object must be to excite those organs which are weak and torpid; whilst the organs or structures that manifest too great a degree of activity must be repressed and soothed. It is in this way that we establish an equilibrium in the actions of the animal system, and, in a manner, place the various organs in a condition which enables them to aid each other in gaining their healthy conditions. We pass over the sections on the "*modifications relative to professions;*" the "*modifications relative to habits;*" and those relative to "*appetites.*" In relation to the general indications derived from the "*causes* of *diseases*," our author shews himself again *tout a fait* Broussaian. The "physiological doctrine" he says, "teaches us to neglect certain occult hypothetical causes, that occupied so great a share of the attention of our predecessors, and which they attempted to reduce by the most injurious remedies. Hence the ideas of the pretended alteration of humours; of a peculiar gouty, rheumatic, or herpetic disposition, to which acute and chronic affections of the viscera were so often attributed, *have been totally exploded.* Here M. Begin calculates without his host. We are quite certain that there are still some physicians who do not believe that gout, rheumatism, and herpes are mere sympathetic phenomena of a *gastro-enteritis;* and who "dare to maintain," that there exists very peculiar and, they confess, occult causes in the animal organization, intimately concerned in the production of these affections. We have not space, in this article, to enter into a discussion on this subject; for we have as yet advanced but a short distance into our author's work; and there are a multitude of topics remaining, to which we may more profitably direct the readers' attention.

When speaking of the "modifications in the general indications of treatment arises from the seat of diseases," the author repeats a favourite maxim of Broussais, which is by no means generally applicable. "In inflammations of the membranes" he says, "we are not to carry general bleeding to the same extent as in those of the parenchymata." Does it require less direct depletion in inflammation of the pleura than in inflammation of the substance of the beings? The reverse is generally the case. Does acute inflamation of the bladder, of the stomach, and of the peritoneum not allow us to carry venesection to the same extent as is required in inflammation of the kidney, liver, or pancreas? Broussais has given us another maxim of this kind, however, to which we readily give our assent; namely, that blood-letting does not in general exert the same beneficial influence over inflammation of the *mucous membranes*, nor can it usually be carried to the extent, as in inflammation of the parenchymata and serous membranes.

M. Begin ridicules the idea, that the different stages of diseases require a variation in the treatment. "Since the time of Hippocrates," he says, "to the present day physicians have generally advocated the vague and irrational precept, that the treatment of diseases is to vary according to their periods. No rigid mind can read, without disgust, what the ORTOLOGISTS have written on this subject." We confess, readily, that we are among those who advocate this ontological, and to M. Begin, disgusting heresy. We were taught to pay especial attention to the stages of diseases, as all-important circumstances for the regulation of our therapeutic efforts; and a course of upwards of twenty years of experience, has deeply impressed us with the conviction, that in teaching others, we could not well inculcate a more useful principle than the necessity of paying a close attention to successive periods of acute maladies. We should deem the physician a very bold and ignorant practitioner, who would treat the stages of *excitement* and *collapse* of a case of malignant scarlatina, for instance, with the same class of remedies. The physician who would give wine, carbonate of ammonia, and other articles of this kind, in the stage of excitement of typhus, confluent small-pox, scarlatina maligna, &c., would be a most excellent contributor to the undertaker, but a most execrable counsellor to the patient; and yet these very remedies would in all probability constitute the best means for preserving the life of the patient during the last and sinking stage of the disease. We do not mean to dispute the well established position, that the most certain means for preventing dangerous exhaustion of the vital energies in the advanced stages of acute diseases, is a prompt and energetic employment of the most efficient antiphlogistic measures in the commencement and early period of the disease. But common sense and universal experience is opposed to the continuance of these means where collapse has actually supervened, at least without the concomitant employment of remedies calculated to sustain the vital powers. Our author compares the exhibition of tartarized antimony "in the commencement of pulmonary catarrh, to the vulgar practice of giving alcoholic stimulants, and other heating sudorifics." Upon what facts and experience he is led to regard tartarized antimony as a "*perturbating* agent," analogous in its consequence to "alcoholic substances," require more sagacity than we can pretend to possess. We apprehend that Rassori, Thommasini and some of his own countrymen; particularly Laennec, and Fontancilles, would tell him that this assertion is an evidence either of extraordinary ignorance on his part, in relation to the proper powers of this article; or one of those senseless extravagancies into which zealots of all kinds are apt to be hurried, when descanting on the supreme excellence of their favorite doctrines. The alpha and omega, the whole, almost, of what, in the estimation of a disciple of the physiological school, is useful among therapeutic agents, consists in local and general depletion; mucilaginous diluents, laxative enemata, cold and warm aqueous applications, and the negative one starvation. From the beginning to the end of human maladies, and from the commencement to the *finale* of each disease, it is invariably *leeching*, *emollients*, *water gruel*, *lavements*, and *revulsives*. Most assuredly, these are inestimable, and often indispensable means; but we venture to affirm, that were Mr. Begin placed along side of one of our own well educated practitioners of the west, and contending against the furious attacks of our worst forms of autumnal fevers, he would find that, the ontologist of the back-woods, with his "incendiary means," his calomel and jalap, &c., would furnish no data by which the superior and permanent excellence of the physiological medicine would be illustrated.

In the fifth chapter, the author discusses the general character and modus operandi of our remedial agents. Under the head of "*the secondary or general effects of remedies*," the author makes several observations that require notice. Speaking of the extension of remedial impressions throughout the system, the author observes, correctly no doubt, that "the nerves are the immediate agents through which these transmissions take place. They first carry to the brain the impression received at their extremities, which are thence reflected through all the nervous ramifications, occasioning more energetic actions, either in the whole constitution, or in some of the principal viscera. The sympathies excited by the action of remedies are always elective; that is to say, directed towards one or several organs, whose functions they excite or modify. Their effects vary, either according to the medicated organs which excites them, or to the nature of the impression of the remedy of that organ. Another very important consideration is, that the effects sympathetically produced by remedies, vary according to the inert, or the exalted state of the nervous system, and as the organ on which they operate, is either weakened, irritated, or healthy. If the nervous irritability be diminished by long continued fatigue, the most considerable doses of stimulants will prove harmless. M. Dupuy has ascertained that by dividing the pneumogastric nerves of a horse, he was able to administer such large doses of nux vomica as would inevitably have produced instantaneous death, had not the operation been performed. Should the gastric irritation have subsided, it is expedient to increase the dose of medicines calculated to act by means of the sympathies. If, on the contrary, the stomach be irritated, its sympathetic influence on the other organs is perverted: digitalis ceases to moderate the action of the heart; anxiety, vertigo and giddiness, follow the administration of camphor and of all the other excitants; the vital action of the stomach is exasperated by all those substances, and the sympathies it exhibits prove no more than the sufferings of that viscus." Our author does not deny the absorption of some medicinal agents into the circulation. It is but a few years ago, that a learned professor in this country asserted, that "no one who has kept pace with the progress of our science, believes in the absorption of any remedies." At the present time, the application of this sentiment may with justice be reversed; for we may confidently assert, that there are few, if any, whose opinions are worth regarding, who do not now believe that many medical agents act upon the organization through the medium of the blood.

We are told by the author, that although when opium is taken into the stomach in tetanus and other similar nervous affections, its effects are often very trifling, or scarcely perceptible, though given in enormous doses; yet when the same article "is injected into the veins," in the same disease, it will produce its usual narcotic effects. He ascribes the inefficiency of the medicine in the former mode of exhibition, to the powerful assimilating powers of the stomach, by which the remedy is decomposed, or in some way or other rendered less active. "It has been long established, he says, that when thrown into the rectum, in cases of nervous irritations, liquid opium will produce more effect than when given through the mouth. These facts seem to indicate that the absorption of medicines is sometimes indispensible for their operation."

The specific tendency of certain medicines to operate on certain organs or structures, is especially worthy of notice in a physiological point of view. This tendency is particularly illustrated by the effects that medicines have when injected into the veins. Thus emetics, purgatives, narcotics, diuretics, &c. always affect exactly the same organs when introduced into the circulation by artificial means, as if they had been introduced into the stomach. Dr. E. Hale asserts that he injected castor oil into his own veins, and that the consequences were the same as if it had been introduced into the stomach. We are much inclined to believe that too little attention is generally paid to the skin, the rectum, and the lungs, as avenues for the introduction of remedial impressions. When we reflect how generally the sensibility of the mucous membrane of the stomach is deranged; and how apt the action of the digestive powers of the stomach must be to alter the composition of medicinal substances—particularly those derived from the vegetable kingdom, we cannot but conclude that the influence of remedies must be much more liable to be weakened, or altered from their physiological effects, when taken into the stomach, than when applied to the skin with or without its epidermis, or injected into the rectum, or inhaled into the lungs. We have recently had an interesting example of the value of varying the mode of remedial application. A weak and delicate female, who suffered much from anomalous gastric pains of a neuralgic character, made several attempts to take opium in various forms; but it always produced so much general distress, and disturbance of the brain that she refused to take it again. She consented, however, to its external use, and we accordingly removed the cuticle by means of a blister about the size of a dollar, and applied a grain of morphia to the raw surface. The effects were delightful. The anodyne influence of the medicine were fully obtained, without the slightest inconvenience either during its operation or afterwards.

The seventh chapter treats of "*the classification of therapeutic actions.*" According to the author, "the excitement and depression of vital actions constitute the only means we possess of modifying the living organs in the state of disease." He affirms that there are no specific diseases, and consequently no specific remedies; but he seems to distrust his own opinion on this point, and at all events, offers but very lame arguments against the "ontological specificity" in pathology and therapeutics. "If," says the author, "the ontological specificity is to be banished from pathology and therapeutics, we should not overlook those facts on which it appeared to rest its foundation. Experience and daily observation teach us that among physical bodies, some operate especially on certain organs or tissues, which they always stimulate in such a manner as to produce constantly the same effects. Thus vaccine virus, the matter of syphilitic ulcers, masniati emanating from subjects affected with rubeola, variola, &c. are as many irritating causes which generally occasion phenomena identically the same in all cases. This property of constantly acting in the same manner, and on the same parts or organs is also met with in some medicinal substances. Sulphur, for instance, seems specifically to stimulate the skin; digitalis moderates the action of the heart; narcotics act generally in the same manner, in all cases, on the nervous system; mercury on the salivary glands, &c. Here we must evidently acknowledge a speciality of action; in other words, an irritation or debility constantly produced in the same manner, and on the same parts, *but there is no specific effect in them;* that is to say, those substances do not produce modifications, which would be neither excitement nor debility in the parts which are the seat of the stimulations occasioned by them." The sentiments involved in the latter part of this long extract, are singular specimens of theoretical absurdities and nonsense. It appears to us that it would scarcely be treating the common sense of our readers with due respect, to offer them any arguments in refutation of these assertions. We are gravely told, that though there is a "speciality of action," there is no specific power in the articles mentioned; because, forsooth, they all produce an increase of excitement! Mercury has no specific powers: for although it produces an effect which no other known agent is capable of producing, it agrees with most other agents in being an *excitant!* But this is not all. To our astonishment, we find that our notion as to the reality of *specific diseases*, is but a mere ontological chimera! Measles, scarlatina, small-pox, and so forth, do not differ essentially or specifically from intermitting fever, gout, and hooping-cough! They are, all, nothing more than diverse manifestations of *gastro-enteritis:* differing only in the *greater* or *less* degree of local gastro-enteric irritation upon which they depend. According to this point in the Broussaian doctrine, a patient might successively pass through almost the whole catalogue of diseases, in the progress of a case of gastro-enteritis, from its use, to its acme and declension. But we will not waste our time in dwelling upon assertions and opinions repugnant to common sense and observation.

The *Second Book* commences with the consideration of the "*Debilitating means of predication.*"

The author observes—and correctly—that the indication for diminishing the vital actions is that which most frequently occurs in practice. He divides the means for fulfilling this general indication, into two orders. The first embraces the measures proper for removing all stimulants not necessary to the maintenance of life, or for lessening their action on the system; and sanguineous depletion. The second order for debilitating means, consists of those medicinal agents, which experience has shewn to be capable of diminishing the vital actions—such as nitre, digitalis, emollients, cold applications, &c. The observations of our author on the therapeutic application of cold, are in general judicious, and accordant with the experience of the profession. "In almost all cases, he says, to avoid too sudden and too violent a shock of intense cold, it may be well to precede their employment by the application of water, the temperature of which will be lessened, until ice itself be used." From our own observations we are satisfied that this rule deserves particular observance, especially when cold is applied in the diseases of delicate and irritable subjects. In local inflammations great benefit may sometimes be derived from the application of intense cold. The author states, on the authority of Reuss, that we may often arrest the inflammation in the commencement of whit low, by holding the finger in melting ice, or very cold water and repeating it several times during the day. During the preceding winter we witnessed two examples of the complete success of this practice.

Local blood-letting, by *leeches* is the *remedium magnum* of the "physiological medicine;" and, it must be admitted, that we have very few means, that can rank in usefulness with capillary depletion, in diseases attended with local inflammations. The following remarks, on the hemorrhage which is apt to occur from the leech bites is particularly worthy of attention. An inattention to this circumstance, has, within our own observation, cost the life of a robust and charming infant. "There is no puncture of the size and depth of leech bites which can discharge so much blood. It has sometimes happened that when left to themselves they have occasioned serious and even fatal hemorrhages. Robust men, in the prime of life have been reduced to such a degree of paleness and debility that they appeared on the verge of dissolution. I have seen the blood run through a double matrass and a straw bed, from not more than twenty-five leech bites on the abdomen. Young people do not resist so well as others, hemorrhages of this nature. M. Pelletan relates the case of a child six years old, who died from the loss of blood, after the application of six leeches to the chest; the female attendants had done no more than wash off the blood as it flowed." We have already said that a similar accident occured in our own practice. Fifteen leeches were applied to the temples of a child about eight months old affected with arachnites. After the leeching was over, which was late in the evening, the child was laid in its cradle. Towards morning, the mother found the child, pale, cold, and evidently in a dying condition. We were sent for; and on examination found the pillow and bed upon which the little patients' head rested completely saturated with blood. The child was expiring from exhaustation. "Cupping and scarifications occasion analogous phenomena; with this remarkable difference, however, that the loss of blood is not so great, nor so easily kept up and continued, and that the cutaneous irritation either during or after the operation is more intense than that resulting from leeches. In a word the former process is less depletive and more stimulant than the latter."

It should be observed, however, that very little or no advantage will in general result from capillary blood-letting, when the inflammation is intense, the patient robust and plethoric, and the action of the heart and arteries vehement. Under such circumstances general efficient blood-letting, is an indispensable preliminary measure to the employment of leeching or cupping. So long as the general momentum of the circulation continues to be considerable, the application of leeches to the afflicted part, will "almost inevitably increase the impetus of the blood to it, and augment the local affection." The irritation produced by the leech bites, is sometimes so great as to cause an injurious degree of inflammation on the surface to which the leeches are applied, "The skin becomes red, and somewhat tumefied, and each puncture forms the centre of an inflammatory disc, which, uniting with the adjacent ones, covers the whole surface of the part with an inflammatory crust, more or less extensive." The inflammation thus excited, is apt, when the inflammatory diatheses is considerable, to aggravate the general febrile symptoms, and to become the source of no small degree of inconvenience and injury. "It is, hence, always necessary that the letting out of blood from the capillaries be sufficiently considerable to counterbalance and prevent the nflammatory process which is apt to follow the operation. This precept admits of no exception. When the disease is intense, and the number of leeches is not sufficient it is not uncommon for them to prove inefficacious, and even to increase the symptoms. We must therefore guard against this disappointment, by applying them in such quantity that they will prevent the subsequent stimulation which may be occasioned by the punctures."

Many very correct and useful observations are made by the author touching the therapeutic employment of capillary bleeding; but we find also in this part of the work, as indeed throughout, much in relation to the pathology of diseases that is wholly inadmissible, and untenable on any resonable grounds. Passing over the third and fourth chapters of this section of our author's work, in which are considered, "*debilitating medications applied to the organs of the senses*, and those "applicable to the genito-urinary organs," we stop to cull a few observations from the fifth chapter—"*On debilitating medications applied to the organs of respiration.*"

"Clinical observations" says M. Begin, "demonstrate that the most intense and obstinate pneumoniæ, and consequently the most dangerous, *frequently* succeed a violent gastro-enteritis, during which the irritation sympathetically carried to the lungs, becomes predominant in them, whilst that of the digestive organs thereby disappears." That pneumonia may sometimes occur in this way, is not at all improbable; but the assertion that this is a *frequent* occurrence, is most assuredly not warranted by observation. We are perfectly aware that slow pulmonic irritation passing ultimately into inflammation and disorganization may, and, indeed, not unfrequently does, arise in consequence of continued gastro-intestinal irritation; and hence the frequent development of phthisis pulmonalis in scrofulous habits, from dispeptic irritation of the stomach. The sympathy between the lungs and skin, appears however, to be much stronger than that which subsists between the former organ and the alimentary canal. It is on this account that cutaneous irritations are so apt to manifest an influence on the mucous membrane of the lungs;" that the repulsion of acute and chronic eruptions of the skin so frequently cause disease of the respiratory organs; and that after violent excitations of the vessels of the surface, or during an active state of the cutaneous exhalents the sudden application of cold is so liable to give immediate rise to serious pectoral affections.

The means by which we may "debilitate the organs of respiration," in other words the means by which we may lessen the vascular inflammatory excitement of the organs, as stated by the author are, "1. lenient drinks. 2. evacuations of blood; 3. emollient fumigations, and various substances which are sometimes used in the form of vapour; 4. calming and sedative applications on the external surface of the chest;" and to these we would add counter irritating revulsives; and various sedative remedies, such as tartar emetic, digitalis, nitre and cathartics, &c. In speaking of emollient diluents, the author, observes: "we must here notice the errors into which empirics have generally fallen, in prescribing *hot* pectoral emollients. These liquids should not be given cold, even in the most acute phlegmasiæ." In this remark, we entirely agree with our author, only, that we should not regard it as an evidence of empiricism to differ from us in this opinion. The author seems to think very highly of the "broth of frogs and snails," as an emollient drink for this purpose; in this country we apprehend, it would be ranked by most patients under the head of *emetics*.

The sixth chapter is devoted to the consideration "of debilitating medications applied to the alimentary canal." As irritation of the mucous membrane of the alimentary canal, is, according to the physiological doctrine, the *fons et origo* of, perhaps, nine tenths of all human maladies, "the debilitating medications" of this structure constitutes incomparably the most important subject in the therapeutics of this school. Speaking of the extensive sympathies of the digestive tube, the author observes, that the portions of this organ that are above the cardia, and below the cœcum, "are under the laws common to the whole organization, and present no prominent character with respect to the sympathies which they excite." In relation to the part below the cœcum, this observation does not appear to us entirely correct. Irritation of the rectum from ascarides and other causes, is apt to give rise to various sympathetic phenomena, manifesting very lively susceptibility, and range of sympathetic relations. Unquestionably, however, the mucous membrane of the stomach and small intestines predominate, in this respect, over every other organ and structure of the animal system. The stomach, especially, appears, in its susceptibilities of receiving and propagating sympathetic excitements, like a subordinate *sensorium commune*. In addition to these obvious physiological properties of the mucous membrane of the alimentary canal, Broussais, has ascribed to it another one which it was necessary to assume, in order to give a plausible consistency to the gastro-enteritic etiology of certain febrile affections. He asserts, namely, that all morbific impressions made on any part of the body, with sufficient force to produce fever, are in the first place conveyed to the brain, and thence reflected to be "repeated" and firmly established in the mucous membrane of the stomach and small intestines. When a patient, for instance, inoculated with small pox virus, the irritation which, after a few days, is produced in the point where the virus was inserted, is conveyed by the nerves to the brain; the brain then reflects this irritation upon the mucous membrane of the stomach and intestines, where it speedily establishes a high degree of capillary irritation or inflammation. Thus gastro-enteric inflammation now radiates its irritations throughout the system, rouses the heart and arteries into febrile reaction, and, after some time, a kind of critical metastasis of the irritation takes place from the alimentary canal to the skin, where it causes the variolous eruption. Cold, in giving rise to ordinary synochal fever, is said to produce an impression upon the surface of the body. This impression is conveyed to the brain, and thence reflected upon the mucous membrane of the stomach and bowels, where it establishes a certain degree of inflammation, which then becomes the immediate source of all the febrile phenomena. Now this supposed connexion between the brain and the intestinal mucous membrane,—the assertion, that all irritations made on the periphery of the nervous system, after having been transmitted to the brain, are reflected and *repeated* in the gastro-mucous membrane, passing almost immediately into inflammation, is nothing more than a gratuitous assumption,—a physiological dogma, adopted, manifestly, as a principle necessary to the consistent construction of the gastro-enteritic hypothesis. We do not mean to deny that, impressions made on any part of the body are conveyed to the brain, and that this organ reflects them throughout the nervous system. But we demur at the assertion, that these reflected cerebral irritations are exclusively directed upon the mucous membrane of the stomach and bowels. We presume that every organ and structure is susceptible of receiving them; and that the part in which they chiefly manifest themselves, depends on the accidental debility or state of irritability of this or that organ or structure. We would maintain also, that impressions thus reflected, very rarely give rise to inflammation in a direct manner; but that they cause, simply, more or less functional disorder, or weakness and irritability in the part, which, on the supervention of the febrile reaction, may readily pass into inflammation. But we have not space to pursue these observations. The author gives "the following debilitating applications as applicable to the digestive canal: 1st. Abstinence from all excitants, both in food and liquids; 2d. Drinks capable of lessening vital action in the mucous membrane of the stomach; 3d. Emollient applications to the abdomen; 4th. Local, capillary blood-letting; 5th. Cold, both externally and internally." These, then, in conjunction with general blood-letting, constitute the therapeutic means for combatting typhus, bilious, synochal, yellow, and all other forms of fever, and which, our author urges, to the entire exclusion of "that routinous polypharmacy," such as purgatives, emetics, calomel, antimonials, &c. We certainly do not mean to deprecate the "debilitating medicines" enumerated by our author; on the contrary, we are entirely convinced that they are among the best curative means we possess, and in many cases, are indispensible to the successful management of the disease in question. We need not adopt the Broussaian etiology of febrile diseases, to give full importance to these remediate measures which they unquestionably possess. In many instances of every form of fever, more or less inflammation of the mucous membrane of the stomach and bowels is apt to occur in the course of the malady. Not unfrequently we find, in the ordinary autumnal and winter fevers, the abdomen to become distended, tense, and painful to pressure, accompanied with watery stools, of a reddish and flocculent appearance, at the same time that the skin is dry, harsh, and very hot, and the margin of the tongue red and raw. Here, most assuredly, leeching and revulsives to the abdomen, with mucilaginous drinks, &c., and an abstinence from active purgatives, and the usual saline antiphlogestic preparations, is to be regarded as the appropriate course of remedial management.

Our author thinks that cold, both internally and externally, has not been sufficiently resorted to by physicians in gastro-enteritis, peritonitis, and other abdominal affections. "The most violent forms of gastro-enteritis have often been successfully treated by cold drinks. Crillo, of Naples, gave two pounds of water every two hours, and prescribed a most severe diet in cases of fever; he persevered in this treatment until the appetite returned, and indicated a sensible improvement. Some German physicians have related numerous cases of fevers successfully treated by cold drinks. In cases of actual inflammation of the mucous membrane of the stomach, we are disposed to place no small degree of reliance on the use of cold water. Some ten months ago, we attended a black man, laboring under acute gastritis, attended with the ordinary symptoms of this affection. We directed him a wine glass full of ice-water, every half hour. He expressed himself greatly relieved by this indulgence, and it evidently exerted a very excellent effect upon the inflamed organ.

That the therapeutic principles, and particular remedial applications inculcated by Broussais and his followers, are very judicious in reference to *actual inflammation* of the mucous membrane of the alimentary canal, no one will dispute. The question at issue, however, is whether the same means are applicable to fevers generally, to the exclusion of purgatives, emetics, calomel, &c., under the assumption that these affections are only different modifications of gastro-enteric inflammation. "What authors have called bilious or gastric fevers," says M. Begin, "are nothing else than more or less violent irritations of the stomach, accompanied with secondary excitements of the liver; and the means recommended against gastritis, are sanctioned by daily experience. All laxatives are to be condemned in such cases. None but blind humorists could have established those barbarous indications consisting in the expulsion of bile, mucous, saburral obstructions, and other matter. Let candid physicians compare the result of the phisiological method, with those obtained by that perturbating incendiary or evacuating treatment so generally employed in fevers, and let them decide." If our author be correct in these remarks, it may be considered as a most extraordinary circumstance, that the physicians of this country, and particularly our western brethren, who prescribe so freely all these "incendiary evacuations;" nay even calomel and jalap, and perchance tartar emetic, salts, and nitre, do nevertheless cure their patients, on a fair comparison, as often at least, and we venture to say oftener, than the most rigid advocates for the exclusive employment of emollients, leeches, fomentations, &c., can claim.

Even admitting, however, that the gastro-intestinal mucous membrane is always peculiarly irritable and prone to inflammation, in fevers of this kind, it appears to us that there is much more reason to apprehend the production of inflammation from the constant and long-continued impressions of the acrid and vitiated secretions collected in the bowels, than from the occasional transient excitement caused by a purgative. We constantly read in the accounts of the post-mortem examinations published in proof of the "physiological doctrine," that the contents of the bowels were filled with very offensive matter—pungent and acrid (*Bouillant*) fluids. But we are told that these acrid secretions are quite harmless. "Nature," says an ingenuous American disciple of this school, "finds but little difficulty in getting rid of them. The product of a certain condition, these secretions bear most generally a relation to that condition, whether it be natural or pathological; and when the two are in the same relationship, the secretions, however morbid, cannot react injuriously on the organs." But how, we would ask, can the vitiated secretion of bile by the liver, be in relation with the mucous membrane of the bowels? We might as well say that the saliva is in relation to the conjunctiva of the eyes in a case of catarrh. Altogether, this pretended relation between the secreting surface in a state of disease, and its secretions, appears to us very questionable. Be this as it may, however, we must observe, in relation to the point under consideration, that the acrid contents of the bowels is by no means wholly the product of secretion. The fluids, taken in and mixed up with those furnished by secretion, not being subjected to the ordinary antiseptic powers of the digestive organs, readily enter into chemical changes, giving rise to various acrid and irritating combinations, which cannot be in relation with the mucous membrane of the alimentary canal. But we have already extended this article to a greater length than we at first intended; and, although there are materials remaining in the work before us for much discussion and criticism, we find ourselves obliged to stop short, as the translation is wretchedly executed.

